# The Flow of Learning

**DOI:** 10.5964/ejop.v14i2.1660

**Published:** 2018-06-19

**Authors:** Maciej Karwowski

**Affiliations:** a University of Wrocław, Wrocław, Poland

Saying that learning is complex is akin to saying nothing. Apparently, learning is not only dynamic and interactional, but also pretty challenging to understand – it is by no means an easy task to untangle all its nuances and idiosyncrasies. Thus, it is not accidental that scholars sometimes compare learning to a river (see Alexander, Schallert, & Reynolds, 2009). Indeed, as they note (see Alexander et al., 2009, p. 178), learning involves change, is inevitable, changeable at different points, and incredibly dynamic. Precisely as a river, one may agree. But yet – still in line with rivers – learning can be resisted or could be disadvantageous: after all, if learning is change and change breaks our comfort zone, then learning could bring negative outcomes as well.

Therefore, although laypeople and professionals tend to perceive learning as ubiquitously positive, it is not necessarily always the case. Learning helped our antecessors survive on savannahs and keeps helping us function today. Today’s learning, however, especially the institutionalized one, too often equates with a static, prior-knowledge-based process of memorization rather than a dynamic transformation into skills and individually meaningful discoveries. Can learning be inventive at all, though? Or, perhaps, is any learning creative by definition? While it is hardly possible to create something meaningful if someone lacks the necessary knowledge, the question arises to what extent the process of learning itself may be framed as creative. If learning is a river, is creative learning a waterfall? These questions are not trivial; after all, school systems are often perceived as being responsible for hindering students’ individuality rather than inspiring it and, even if such an attitude is more ideological than empirical, it is too widely spread to be ignored (Robinson, 2001).

The conviction that school and creativity are opposites seems to stem from three sources – each of profoundly naïve character. The first is a clash between the romantic myth of (a supposedly always creative) childhood and the bureaucratized system schools belong to. Many people believe that children are creative by nature, and schools (as instruments of secondary socialization) are anti-creative by their very character. The second source is informed by the shape of curricula and unsystematic observation of everyday school realities. Indeed, practicing algorithms takes up a lot of space in every classroom around the world, likely more space than stimulating curiosity. To be a little bit ironic, one may say that people usually ask questions if they want to get to know something. This rule works poorly, however, if these people teach. Teachers ask questions in response to which they know and want to hear the expected answer. The third source that informs broad public’s imagination are biographies of creators: many Nobel Prize laureates were average students in school, at best, or they remembered their school education as a nightmare. Mark Twain, one of the greatest writers of all times, is credited with a saying that he has “never let [his] schooling interfere with [his] education” (as quoted in Harnsberger, 1972, p. 553). These words perfectly capture the attitude many people have towards school. Briefly: schools have bad reputation.

Is it as bad as critics say, though? Apart from the brilliant creators who did poorly at school, there are others who did excellently: Sigmund Freud, John Locke, August Comte, or Karen Horney, were great students (Simonton, 2002). Similarly, although studies that explore links between creativity and school achievement bring diverse results, they do not support the fact that the most innovative thinkers do poorly at school. In the most extensive meta-analysis conducted so far (Gajda, Karwowski, & Beghetto, 2017), the link between creative abilities and school achievement was weak but positive (*r* = .22). Therefore, current knowledge allows for two, even if premature, conclusions. First, creative students generally do well at school. That’s not too surprising – after all, creative abilities are cognitive abilities – they naturally inform learning. Second, while some original students indeed underachieve at school, it is caused by their lack of motivation, or higher nonconformity rather than creative mental skills *per se*.

## Is Learning Creative?

Previous theorizing on creative learning (e.g., Jeffrey, 2006; Lucas, 2001; Treffinger et al., 1983) criticized school curricula and emphasized the limitations of algorithmic, exclusively intelligence-focused teaching (e.g., Lucas, 2001). Other models equated creative learning with problem-solving (e.g., Treffinger et al., 1983). Thus, these models focused either on intrapersonal mechanisms necessary for learning (students’ traits or cognitive processes) or systemic conditions that support or hinder creativity at school. Less attention was devoted to class ethnography in this context (for an exception see Lucas, 2001) or the role of student-student or student-teacher interaction as a vehicle for creative understanding.

To address this gap, Beghetto (2016) has recently posited that, in the school context, creativity should be analyzed as being involved in the process of learning, but also that learning itself can be creative. Students come up with new and personally meaningful ideas within the context of school subjects. They also share their thoughts and contribute to the learning and understanding of their peers.

What we need to understand is the role played by students’ creativity (their abilities) but also the creativity of the process of internalizing, organizing, and transforming knowledge for the effectiveness of learning. Therefore, scholars approach creativity in learning from two perspectives: differential and processual. The former searches for an answer to the question of whether and to what extent more creative individuals are better or worse students than their less creative peers. The latter searches for discriminants of the original character of learning and explores in what way knowledge is restructured, integrated, and individually interpreted during the process of school-based learning (Anderson et al., 2001; Lucas, 2001). Hence, the process of learning is a particular case of a creative process during which students combine their previous knowledge with new information in a way that is unique for them (Mumford et al., 2012; Sawyer, 2012). Such a process of combining new and unclear content with already acquired knowledge is the core of a creative process. Too little stock of expertise or a lack of creative self-beliefs make it impossible to occur.

## Creative Self-Beliefs (CSB) in the Learning Process

What people think about their skills matters. But our understanding of the mechanisms played by different self-beliefs, including those related to creativity, is still imperfect. Deepening the knowledge about CSBs structure and their interrelations might have profound practical consequences, e.g., for the development of new interventions that strengthen CSBs and, through this, improve students’ functioning.

Why are self-beliefs important for understanding the creative process in general and creative learning in particular? A new theoretical model of creativity as an agentic action (CBAA, see Karwowski & Beghetto, in press) posits creative self-efficacy to mediate and valuing creativity to moderate the relationship between potential and achievement. In other words, this model makes self-beliefs vital factors responsible for mechanisms of effective functioning. The self-beliefs' role, however, is not limited to creative behavior, it informs the process of learning as well.

Creative learning involves sharing new understandings with other students and the teacher. Revealing the new understanding in a classroom forum marks the transfer from intra- to the inter-psychological sphere, because it is applicable not just to the student him- or herself, but also his or her social environment. It requires, however, a sense of agency and competence. For a new idea to turn into a creative input in the current knowledge of others, the teacher, as well as the remaining students, must undertake an attempt to understand it. In being confronted with such a surprising idea, the teacher does not always know how to behave – ignore the idea and consider it a pure misunderstanding of the theme, or make an attempt to understand it and risk disrupting the lesson? If comprehension difficulty is the reason for rejecting the student’s idea, his or her potential and the entire creative process fade, which in consequence has a negative influence on the process of learning and undertaking further attempts at sharing ideas with others. When other students consider the idea to be both coherent and new, it may become an input for their understanding. Consequently, creative learning becomes an interpersonal rather than intrapersonal act.

## Concluding Thoughts

If learning is a river, so is creative learning – maybe an even more tumultuous river. To follow this river means to follow its flow, and monitoring flow requires a dynamic approach. Learning is multidimensional, it depends on self-efficacy, emotions, and is highly situation-dependent. It is impossible to fully capture it using traditional psychometrics or relying on self-report measures. Dynamic processes require dynamic methodologies. Empirical synergies of micro-longitudinal designs with experimental procedures, naturalistic (yet rigorous) observations strengthened by interviews or ethnographic visits in the classroom – all of them may be helpful in untangling the mosaic of intra-psychological and social subprocesses engaged by learning.
